# Preventing interpersonal violence in Panama: is a parenting intervention developed in Australia culturally appropriate?

**DOI:** 10.1007/s00038-016-0874-0

**Published:** 2016-08-30

**Authors:** Anilena Mejia, Fiona Ulph, Rachel Calam

**Affiliations:** 1 Instituto de Investigaciones Científicas y Servicios de Alta Tecnología INDICASAT AIP, Panama City, Panama; 2 Division of Psychology and Mental Health, School of Health Sciences, Zochonis Building, Brunswick Street, The University of Manchester, Manchester, M13 9PL UK

**Keywords:** Parenting interventions, Panama, Australia, Triple P, Qualitative, Violence prevention

## Abstract

**Objectives:**

To explore cultural appropriateness of a transported parenting intervention in Panama.

**Methods:**

Panamanian parents (*n* = 25) were interviewed after participation in an Australian parenting intervention. A thematic analysis was conducted to interpret qualitative data.

**Results:**

Three themes emerged; cultural context, appropriateness of the intervention, and development of support networks. In terms of cultural context, parents described economic difficulties, living in a dangerous world, struggling to balance parenting and work, and using aggressive communication patterns. In terms of appropriateness of the intervention, they rated materials as appropriate, although suggested modifications to its delivery by including children and teachers in the training. Finally, parents commented that the intervention prompted the development of social networks within their communities.

**Conclusions:**

Overall, parents considered a transported parenting intervention as appropriate to their local needs. This study might be useful to local governments and international funders in charge of deciding whether transporting parenting interventions North to South as a strategy for violence prevention would be respectful of local needs. Our findings cannot be generalized beyond Panama, but the methodology can be replicated to answer this question in other settings.

## Introduction

Interpersonal violence (i.e., youth gang violence, intimate partner violence, child maltreatment, and sexual violence) is among the leading causes of death worldwide (WHO 2014). The 2030 Agenda for Sustainable Development suggests “promoting peaceful and inclusive societies, provide access to justice for all and build effective, accountable and inclusive institutional support at all levels” (United Nations 2015). Thus, there is an urgent need to prevent interpersonal violence universally, inclusively, in interdisciplinary teams and using evidence-based interventions that fit the reality of low-resource settings worldwide.

There is growing evidence that safe, stable, and supportive parenting is essential for preventing interpersonal violence (Stack et al. 2010). Parenting interventions have been designed for reducing childhood aggression (Kazdin 1997) and child maltreatment (Lundahl et al. 2006). However, recent reviews suggest that most of these evidences originate in high-income countries (Knerr et al. 2013; Mejia et al. 2012), with only one rigorous trial in an LMIC identified (Cooper et al. 2009), none assessing the cultural fit in the case of transported interventions, and none conducted as a strategy for violence prevention. In the context of a global violence prevention movement, it is key to establish efficacy and appropriateness of interventions transported North to South.

In 2009, the government of Panama funded a project for evaluating a transported evidence-based parenting intervention as a strategy to prevent interpersonal violence locally. A partnership was developed with academics from the UK to explore the cultural fit and efficacy of one intervention from the Australian Triple P Positive Parenting Program (Sanders 2012). Triple P is a system of interventions for parents of children at different developmental stages (e.g., babies, toddlers, school age children, and adolescents) and with different levels of risk. It uses social learning theory (Bandura 1978) to help parents develop strategies for dealing with difficult behaviour in their children with the rationale of preventing child maltreatment and interpersonal violence later in life. We chose Triple P from the list of recommended evidence-based parenting programs published by the United Nations Office on Drugs and Crime (UNODC 2009a) because of previous evidence of effectiveness in different cultures (e.g., Matsumoto et al. 2007). However, its particular fit to the lived reality of parents in Panama have not been systematically evaluated before. Respecting and acknowledging local cultural values have been recognized as key in the delivery of services (Bernal and Domenech-Rodriguez 2012).

Panama is a relatively small, middle-income country located in Central America with about 3.5 million inhabitants. Similar to the rest of Latin America, official language is Spanish, most of their populations are Roman Catholic, and mothers tend to be the primary caregivers of children. Population is ethnically diverse, with 65 % being mestizos, 12.3 % being indigenous, and 9.2 % being afro-Panamanian. Around 40 % of population lives in poverty (World Bank 2012). Interpersonal violence has been recognized as the second leading cause of mortality (Ministry of Health 2012). The government is, therefore, committed to providing parenting support in all schools and increasing investment in children.

**Fig. 1:**
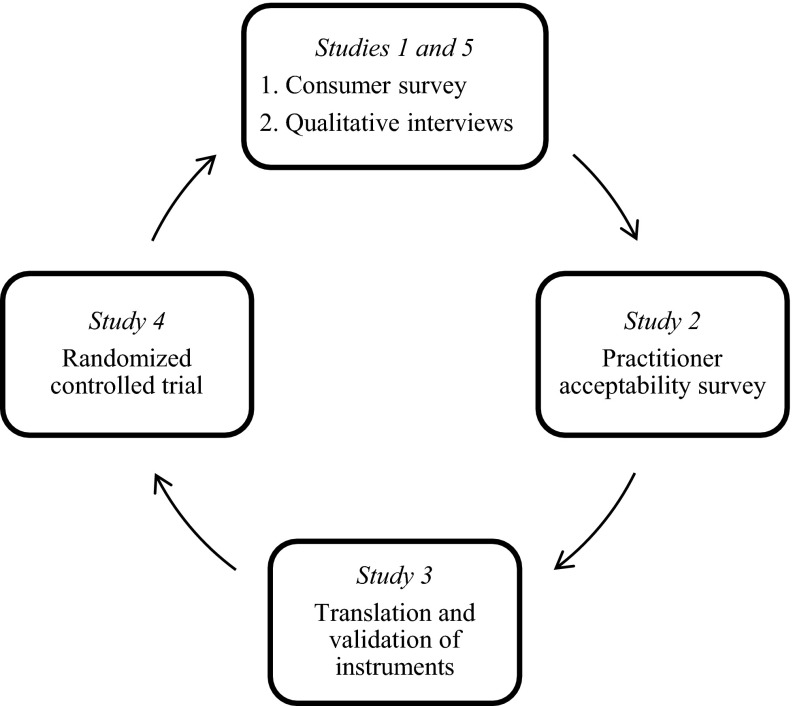
Studies of the project conducted in Panama, 2012

In this project, several studies were conducted using a framework for assessing cultural fit of Triple P before and after an efficacy trial. A similar framework for dissemination and implementation interventions has been previously recommended (UNODC 2009b). The first two studies examined cultural fit from parents’ and practitioners’ perspectives (Mejia et al. 2014a, b). Another two studies translated and validated materials (Mejia et al. 2014c). Subsequently, a randomized controlled trial was conducted (Mejia et al. 2015). The main outcome was child behavioural difficulties, which has previously been recognized as risk factor of interpersonal violence later in life (Loeber and Hay 1997). Figure 1 summarizes the five studies that took place in this project in between 2009 and 2012.

Triple P is a system comprising several interventions for different levels of risk. Only one intervention was chosen for this project; a one-session group format on the topic of dealing with disobedience previously evaluated only in Australia (e.g., Morawska et al. 2011). This specific intervention was chosen, because parents in Panama previously reported preferring brief formats given lack of child care facilities and resources for travel (Mejia et al. 2014a).

This paper describes results from the last study in the project, which re-examined cultural fit of the intervention after delivery, using a qualitative methodology to undertake an in-depth and participant-guided exploration. The aim was to explore, from parents’ perspectives, the appropriateness of this Australian intervention when transported into Panama. It is important to clarify that we were not intending to engage in a cultural adaptation process of the intervention, but rather to assess whether an existing programme from a high-income country, in its original form, was adequate when applied to the reality of this LMIC.

## Methods

### Design

Qualitative data were collected through semi-structured interviews.

### Participants

The trial was conducted with 108 parents (*n* = 54 allocated to the intervention and *n* = 54 allocated to a control group) in Panama between April and November 2012. The present qualitative study took place in November 2012. Participants were recruited from those who participated in the trial, experienced the one-session Triple P intervention, and remained at the last assessment follow-up that took place six months after the intervention. Interviews were conducted after the 6 months follow-up to ensure no contamination of the trial data.

The final sample was composed of 25 parents. These 25 parents were approached by convenience and invited to the interview from the pool of 34 parents that took part in the intervention and remained at the last assessment follow-up 6 months after. All the parents invited agreed to take part and recruitment stopped when thematic saturation was reached (i.e., out of the pool of 34 that remained at the end of the trial, nine parents were not approached due to saturation). Table 1 presents socio-demographic characteristics of the final sample. Mean age (with standard deviations in brackets) is presented in the first two rows, followed by numbers and percentages for the remaining variables.

**Table 1:** Socio-demographic characteristics of parents interviewed in Panama, 2012 (*n* = 25)

	*n* (%)
Age (years)	36.84 (8.32)
Child age (years)	8.21 (1.86)
Child gender	
Male	18 (72)
Female	7 (28)
Relationship to child	
Mother	21 (84)
Stepmother	1 (4)
Grandmother	2 (8)
Aunt	1 (4)
Marital status	
Married	9 (36)
Divorced	1 (4)
Single	5 (20)
Cohabiting	10 (40)
Educational level	
Primary	9 (36)
Some high	7 (28)
Finish high	7 (28)
UG degree	2 (8)
Working status	
Full time	4 (16)
Part time	8 (32)
Looking	3 (12)
From home	1 (4)
Not working	9 (36)
Monthly income (in USD)	
Not revealed	5 (20)
Less than 100	1 (4)
100–249	15 (60)
250–399	4 (16)

### Measures

The interview schedule had questions about (1) parents’ experience in the intervention, (2) changes after, and (3) cultural relevance. The first author designed the schedule in discussions with other authors, including a qualitative research expert. The schedule was piloted with two parents from similar communities to the interviewees. No changes were made after piloting. A copy of the schedule is available upon request.

### Analysis

Interviews took place in Spanish and lasted approximately 30 min. Recordings were transcribed and translated into English simultaneously by the first author. Local expressions and meanings were maintained when possible. Confidential information was replaced with pseudonyms. The first author and second author conducted the analysis in Nvivo v9.

Thematic analysis has been defined as a method for interpreting large sets of data (Rice and Ezzy 1999). Data coding started at the manifest level and deductively. Nevertheless, all codes and the third theme emerged in an inductive manner directly from the data. A hybrid approach for thematic analysis (i.e., themes emerging both inductively and deductively) has previously been recognized as appropriate (Fereday and Muir-Cochrane 2006). The final thematic map was modified iteratively based on team discussions.

## Results

**Fig. 2:**
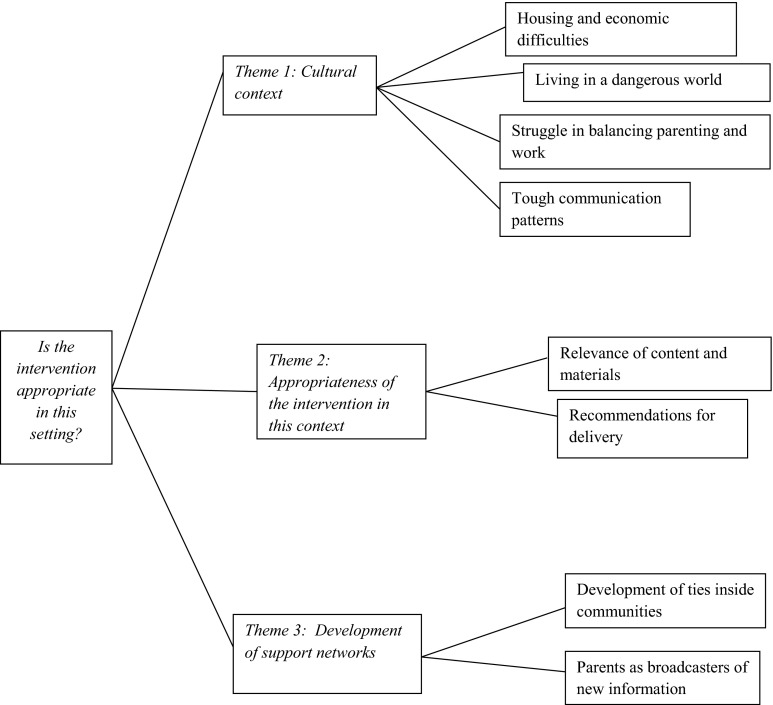
Themes that emerged from interviews to parents in Panama, 2012

Our aim was to explore the cultural context in which Panamanian parents live (theme 1) and appropriateness of the Australian intervention in this context (theme 2). An inductive analysis revealed a third theme concerning the development of social networks. Figure 2 summarizes the main research question that guided the analysis (far left), themes that emerged from the data (centre), and codes that emerged from each theme (far right).

### Theme 1: cultural context

*Economic difficulties* Parents often mentioned stress associated with economic constraints.

Most parents don’t have anything to cook. Some families live in houses with soil floors. That doesn’t allow us to raise our kids as we would like to. (#11, mother; son aged 11)

They described poverty and housing difficulties as obstacles to raising their children, as they would like to. These difficulties were more common for single mothers, as is the case of the participant above.

*Living in a dangerous world* A particularly striking narrative coming spontaneously from parents was experiencing great anxiety due to community violence.

We live in a dangerous neighbourhood with delinquents and drug addicts. I am scared my child will leave home and get involved with them. (#24, mother; son aged 10)

Parents discussed ways for preparing their children to confront this dangerous world, which often involved teaching them to respond aggressively.

They need to be prepared for this dangerous world, so I am teaching him how to defend himself. (#11, uncle; nephew aged 10).

Some other parents said that they approached the issue by overprotecting their children, and thus community violence seemed to be an added stress impacting their parenting style.

*Struggle in balancing parenting and work* Several parents mentioned stress associated with balancing parenting and work, but for many, finding money to eat was above and beyond parenting needs; there was no point in being an affectionate parent if their children could not eat.

It is difficult because I am the economic support. I need to move and look for money. (#22, father; daughter aged 10)

This situation is common in Panama, where parents have to deal with multiple informal jobs (e.g., painting houses) often referred to as “little shrimps” (*camaroncitos* in Spanish) and needing to move, where the jobs are.

*Tough communication patterns* Another particularly striking code was the use of hostile communication strategies, a known risk factor of child maltreatment. Some parents felt ashamed about these patterns and reflected on them as something they would like to change.

I was very explosive. I didn’t have patience. I punched him a lot. (#2, mother; son aged 9)

I spanked him so much that I couldn’t stop. I would make scars on him. (#20, mother; son aged 6)

Others normalized it and said it is part of the Panamanian culture.

Here in Panama we talk and yell. […] It is normal here to spank your kid. (#9, mother; daughter aged 8)

### Theme 2: appropriateness of the intervention in this context

*Relevance of content and materials* Some parents said that similarities between cultures seemed to outweigh differences.

Parents face the same problems everywhere. There are disobedient kids everywhere, so for me the program was very appropriate. (#7, mother; daughter aged 7)

Parents identified with actors in the videos, although there was no physical resemblance.

I liked the videos even though the people didn’t look like us. But what really matters is the content. (#4, mother; daughter aged 6)

Most parents considered that socio-economic differences with parents in videos were superficial and did not impact on their acceptability of the materials.

In the videos they showed families with a higher class, but they still had the same problems. The only difference is that we don’t have money. (#9, mother; girl aged 8)

Overall, most parents suggested that materials were respectful of their cultural reality. However, some parents identified that activities to be implemented at home were not in tune with their reality.

My house is really small and I don’t have space to implement the strategies. Also, giving rewards is difficult when you don’t have money to eat. (#3, mother; boy aged 9)

In addition, they suggested adding training for reducing hostile communication, patterns which are recognized as characteristic to their context.

I yell and I say bad words, and they didn’t in the role-plays. They should show situations more appropriate to the ghetto. (#3, mother; son aged 9)

*Recommendations for delivery* Parents said that the intervention should reach more community members to make a change at the societal level.

I would like to see the program not only in this community, but in every school in the country. (#10, mother; boy aged 11)

Several parents also suggested that the facilitator should also have contact with the children to fully understand difficulties.

The kids should come to see what their parents are learning, because if both change, probably results are going to be faster (#19, mother; daughter aged 11).

The need for a consistent approach involving all actors in the child’s life was also recognized.

Teachers should take part in this program because if we are taking the time, then they should do the same in the school. (#9, mother; girl aged 8)

These examples suggest that parents favoured preventive interventions that targeted different levels at the same time (i.e., the child and the school) allowing the consistency of all actors involved.

### Theme 3: development of social support networks

*Development of ties inside communities* Parents mentioned feeling isolated and wanting some peer guidance on how to do things better.

I see a lot of parents every day and we don’t know each other. The intervention was helpful in this aspect (#7, mother; girl aged 7)

It seems that the intervention helped parents to establish monitoring and motivation networks.

I asked her how she was doing. I also asked if she is using the behaviour chart, and she asked me about my kid. (#1, mother; girl aged 6)

*Parents as broadcasters of new information* The intervention not only reinforced networks inside the group, but also with parents who did not participate.

I shared what I learned with others around my house. I gave them the workbook, and they asked me questions. (#13, mother; boy aged 10)

Some parents appeared to be natural leaders in their communities and broadcasting new information seemed natural to them.

I shared with other mums what I learned, because they spank their kids. I try to talk to all the mums and share all the strategies with them. (#5, grandmother; grandson aged 7; member of church and school board).

These data suggest that parents were motivated to support others and to prevent violence in the community as a whole.

## Discussion

International agencies, civil society organizations, and academic institutions have achieved significant milestones in global violence prevention (Lee et al. 2014). However, there is still a lack of empirical knowledge regarding cultural fit when transporting preventive interventions North to South (Knerr et al. 2013; Mejia et al. 2012). To reduce this research gap, the present study was designed to explore appropriateness of an Australian parenting intervention in Panama.

### How do Panamanian parents describe the context in which they raise their children?

Four contextual factors appeared to affect parenting experience in Panama. The first two were economic constraints and perceiving the world as dangerous, two circumstances not surprising in LMICs. How poverty affects child development through mother–child relationships has been previously described (Takeuchi et al. 1991). However, the effects of community violence on parenting have been less studied. It is well established that community violence is associated with stress (Cooley-Quille et al. 2001) and that stress has an effect on parenting practices (Assel et al. 2002). In research carried out in deprived areas of the US, poverty and community violence were associated with psychological distress in parents, which in turn was associated with less positive parent–adolescent relationships (Gutman et al. 2005). Future empirical studies should examine pathways of perpetuation of interpersonal violence in LMIC through dysfunctional parenting practices due to stress associated with community violence.

A third contextual factor affecting parenting experiences was the struggle to balance parenting and work. Adults in these communities subsist through informal jobs, such as selling mangoes outside schools. From an implementation perspective, parenting interventions could be paired with interventions for tackling poverty, such as capacity building for developing small businesses and microfinance activities (Kim et al. 2007). It is also important to consider the need to implement brief interventions that accommodates the life of those who cannot take time off work (Kinnunen and Mauno 2012).

A fourth factor was the existence of aggressive communication. Research documents the impact of spanking on child development (Ferguson 2013) and the perpetuation of these patterns through inter-generational modelling (Dixon et al. 2005). Culturally sensitive interventions to prevent yelling and spanking in this setting are, therefore, needed.

### Was the knowledge brought from Australia appropriate to Panama?

Although parents felt that the intervention was relevant to their culture, they identified elements that should be incorporated to fit this setting, such as more clear images of abusive communication patterns. Involving teachers and children was also recommended which is consistent with research that suggests a need to intervene in higher level systems through complex interventions to have an impact on the child (Swick and Williams 2006). Further efforts should assess the impact of complex interventions versus brief targeted support and its cost-effectiveness before investing in larger scale rollouts.

Finally, it is important to discuss the value of the last theme on the development of social networks. From an implementation perspective, these data allow us to hypothesize that the impact of an intervention can be maximized if parents become informal or formal broadcasters of new information within communities. This snowball effect should be empirically explored, as it might be a cost-effective dissemination strategy by taking advantage of specific cultural values in the context, in this case, collectivism (Uphoff et al. 2013). For example, a train-the-trainer model might be well received in this setting, in which community members are formally trained to disseminate parenting knowledge within communities and subsequently train others to do so. Train-the-trainer models are commonly used for the dissemination of maternal health interventions (e.g., Segre et al. 2011) and are starting to be more widely used in the field of parenting interventions (e.g., McLenna et al. 2009).

### Strengths and limitations

The sample was composed of a diverse range of parents from different communities, including a father and a grandmother. The analysis was carried out by a team of experts as recommended (Lincoln and Guba 1985). However, parents who received the intervention but dropped out before the 6-month follow-up were not accessible. Future studies should seek to include these.

### Conclusion

Parenting interventions might offer a path for preventing interpersonal violence in LMICs, but evaluations of their cultural fit and appropriateness are scarce. In this study, parents in Panama provided some feedback regarding the appropriateness of an Australian parenting intervention in their local context. Acknowledging local needs is key before implementation of interventions in new contexts.
